# Characterizing Multimorbidity from Type 2 Diabetes: Insights from Clustering Approaches

**DOI:** 10.1016/j.ecl.2021.05.012

**Published:** 2021-09

**Authors:** Meryem Cicek, James Buckley, Jonathan Pearson-Stuttard, Edward W. Gregg

**Affiliations:** a Department of Primary Care and Public Health, School of Public Health, Imperial College London, Charing Cross Campus, Reynolds Building, St Dunstan's Road, London W6 8RP, UK; b Department of Epidemiology and Biostatistics, School of Public Health, Imperial College London, Medical School Building, St Mary’s Campus, Norfolk Place, London W2 1PG, UK; c MRC Centre for Environment and Health, School of Public Health, Imperial College London, Medical School Building, St Mary’s Campus, Norfolk Place, London W2 1PG, UK

**Keywords:** Type 2 diabetes mellitus, Multimorbidity, Clustering, Comorbidities, Complications, Patterns, Population health, Risk stratification

## Abstract

Patients with type 2 diabetes mellitus (T2DM) often live with and develop multiple co-occurring conditions, namely multimorbidity, with diffuse impacts on clinical care and patient quality of life. However, literature characterizing T2DM-related multimorbidity patterns is limited. This review summarizes the findings from the emerging literature characterizing and quantifying the association of T2DM with multimorbidity clusters. The authors’ findings reveal 3 dominant cluster types appearing in patients with T2DM-related multimorbidity, such as cardiometabolic precursor conditions, vascular conditions, and mental health conditions. The authors recommend that holistic patient care centers around early detection of other comorbidities and consideration of wider risk factors.

## Key points

• Patients with type 2 diabetes mellitus (T2DM) are at high risk of living with multiple co-occurring conditions.
• Clustering studies show common comorbidities in patients with T2DM are hypertension, lipid disorders, cardiovascular-related conditions (eg, coronary heart disease), microvascular conditions, and depression.
• Generally, individuals who are older, who are female, and who live in more deprived areas are at more risk of having T2DM-related multimorbidity.
• Applying clustering insights alongside other approaches can allow for risk stratification and guide implementation of interventions.
• Clinicians should consider the holistic health needs of individuals living with T2DM, particularly surrounding mental health.

## Introduction

### The Challenge of Type 2 Diabetes-Related Multimorbidity

Managing the rising prevalence of chronic conditions is a key challenge for health systems worldwide.^1^ The co-occurrence of 2 or more chronic conditions is known as multimorbidity (Box 1) and is associated with reduced quality of life, impaired functional status, and increased burden on limited health care resources.^2^^,^^3^ Multimorbidity is a growing problem not only for high-income countries (HICs) but also for low- and middle-income countries (LMICs) who make up 77% of global deaths due to non-communicable diseases, of which 85% are premature deaths.^4^ Some pooled prevalence estimates of global multimorbidity reveal that currently HICs have the highest prevalence (37.9%), whereas LMICs follow closely behind (29.7%)^5^ and are expected to increase over the following years.^6^ By 2035, approximately 17% of the UK population is projected to have 4 or more chronic conditions, almost double the prevalence (9.8%) in 2018.^7^ Previous studies have revealed social inequalities in multimorbidity, demonstrating earlier onset of multiple chronic conditions in patients living in the most deprived areas compared with the most affluent.^8^^,^^9^ This global progression in emerging multimorbidity will have widespread implications for patients, health systems, and governments.

Box 1 **Glossary of key terms**

*Comorbidity* refers to a condition that co-occurs with another condition, with implied reference to an index condition.
*Multimorbidity* refers to the co-occurrence of 2 or more chronic conditions. The difference between comorbidity and multimorbidity is that the former requires an index condition to contextualize the condition, whereas the latter does not assign or differentiate importance on any 1 condition but considers the overall state of having multiple co-occurring conditions.
*Concordant comorbidities* are conditions that share a pathophysiologic pathway with the index condition of concern (eg, in the case of T2DM as an index condition, a concordant complication is chronic kidney disease or liver disease).
*Discordant comorbidities* are conditions that do not share a currently known pathophysiologic pathway with the index condition of concern (eg, in the case of T2DM as index condition, a discordant complication is osteoarthritis).
*Cluster analyses* are statistical approaches that aim to assign a set of objects (or data points) into a defined number of groups, known as clusters, so that objects in the same group possess more similar traits than other objects in other groups.

Patients with diabetes are more likely to develop multiple conditions compared with those without diabetes.^10^ This greater risk reflects the fundamental impact that extended exposure to elevated glucose and insulin resistance have on multiple organ systems, most notably through its effects on microvasculature, macrovasculature, and immune response. These effects in people with type 2 diabetes mellitus (T2DM) lead to an approximate doubling of risk for myocardial infarction, a 5-fold increased risk of renal failure, and more than a 10-fold increased risk of amputation and blindness.^11^ However, the impact on multiple conditions is also partly due to declining mortalities, increased life expectancy, and differing trends in cause-specific mortality, leading to a diversification in cause of death and complications.^12^ Thus, even in the face of declining risk of complications over time, people with T2DM live longer to experience more events and more conditions contributing to multimorbidity. The rising number of young people with T2DM is of particular concern, as earlier onset leads to a longer duration of disease and consequently more years lived with a greater risk for developing other conditions.^13^ Early onset of T2DM can lead to a more disruptive natural history and worsened quality of life,^14^ consequently, having adverse societal effects on labor markets and significant productivity losses.^15^^,^^16^ This review aims to explore how the commonly reported clusters of T2DM-related multimorbidity are characterized in the current literature.

### Shifting the Morbidity Discourse

Historically, health systems have viewed single conditions in siloes as opposed to focusing on the holistic health needs of patients. Similarly, research to identify risk factors and effective prevention and treatment approaches has generally focused on single disease processes, posing challenges in the areas of multimorbidity surveillance, effective interventions, and the interface between care pathways. Thus, most health systems are not fully designed nor sufficiently equipped to provide tailored care to patients with multiple conditions.^17^ However, greater awareness and concern about multimorbidity have led to an emerging literature, moving away from simply counting conditions to instead measuring impact on patients and systems.^18^^,^^19^ Therefore, it is essential that multimorbidity research aims to consider all stages of the life course and focus on complex health states where the burden of multiple conditions is greater than the sum of its parts.^20^

**Fig. 1:**
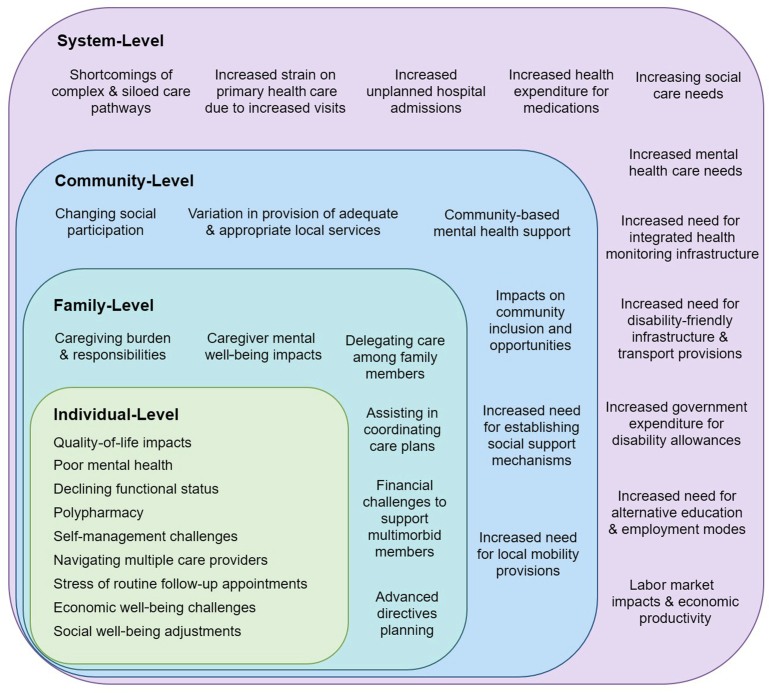
Multilevel impact of T2DM-related multimorbidity.

There are far-reaching impacts of T2DM-related multimorbidity, which have implications not only for patients but also for families, community, and the health system (Fig. 1). Multimorbidity leads to multifaceted demands at the health system level; increased and sustained interactions with health care providers,^3^ adverse events owing to polypharmacy, and nonoptimally integrated care pathways all contribute to the complexity of planning care for multimorbid patients.^21^ This complexity calls for a multitiered approach guided by research that first seeks to understand the patterns of conditions that characterize multimorbidity, the implications for treatments, and how they change over time.

### Development of Type 2 Diabetes Mellitus–Related Multimorbidity

**Fig. 2:**
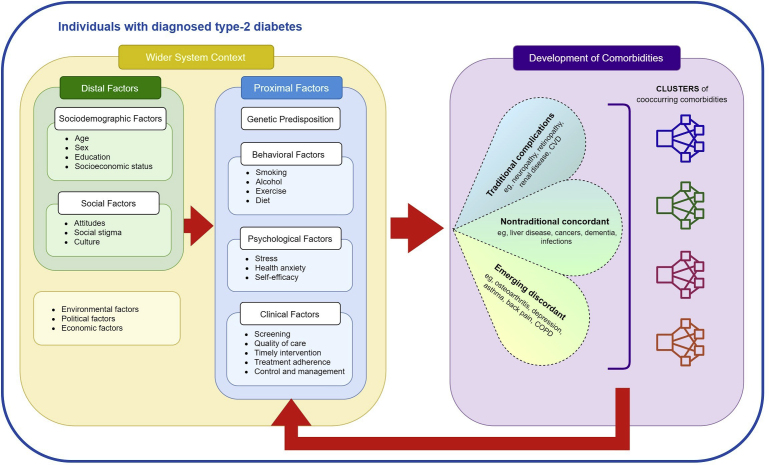
Framework of the development of T2DM-related multimorbidity.

Risk factors of T2DM are shared with other non-communicable diseases, such as vascular conditions and cancers, which can increase the risk of developing further comorbidities. For example, the overlap of risk factors, such as obesity and dyslipidemia, means individuals with T2DM are at higher risk for cardiovascular complications.^22^, ^23^, ^24^ The progression to a multimorbid state is dependent on the contributions of many distal and proximal factors within the wider system context, which can occur at any stage and severity of T2DM (Fig. 2).

Comorbidities contributing to T2DM-related multimorbidity are both systemic yet heterogenous and can be classified as the following:

a. **Traditional** complications include microvascular (eg, retinopathy, nephropathy, neuropathy) and macrovascular (eg, heart disease, stroke, peripheral vascular disease) conditions.^25^
b. **Nontraditional** concordant comorbidities share common risk factors and pathophysiologic pathways but have not traditionally been considered part of T2DM sequelae, such as liver disease and cancers^10^^,^^26^ (see Box 1), in part because they are less specific or have a lower magnitude of association with T2DM compared with traditional complications.
c. **Emerging discordant** comorbidities do not, at present, have a clear etiologic link with T2DM but are found to commonly co-occur, such as depression and asthma, which have garnered increasing interest in the literature because of effects on mortality, morbidity outcomes, and health-related quality of life.^26^^,^^27^

Most of the current literature on T2DM complications has examined comorbidities individually from cohort studies and registries followed over time. However, this body of literature has not fully assessed how multiple conditions form distinct groups and patterns of co-occurrence, and over time. Thus, to inform this synthesis, the authors have conducted a systematic review of the selected studies that have examined the association of T2DM with clusters of multimorbidity. These patterns can be identified using population-based analytical approaches, such as cluster analyses (see Box 1), on T2DM patients.

## Methods

**Fig. 3:**
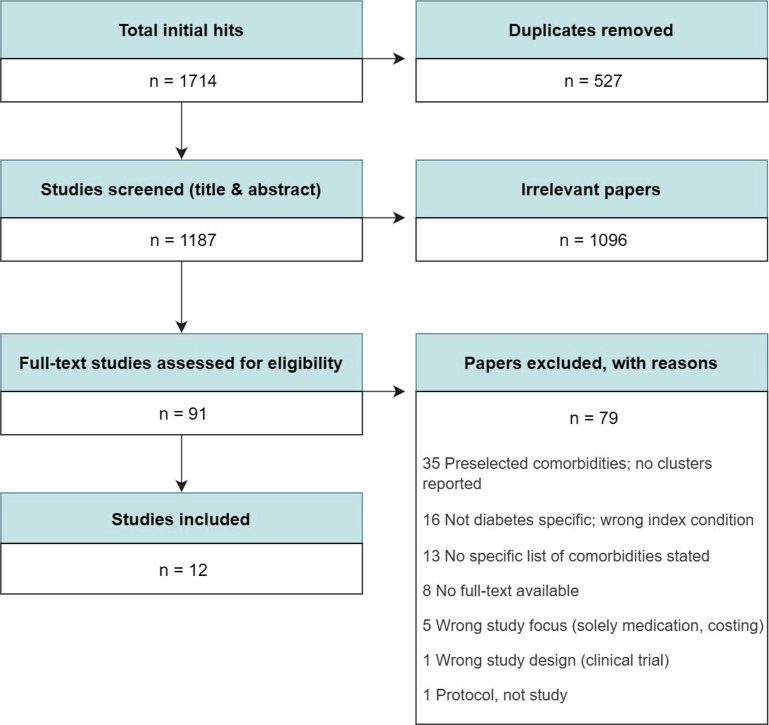
PRISMA inclusion flowchart.

This review aimed to identify population-based observational studies with a focus on T2DM among adults as the index condition, and that produced some type of cluster or grouping in their results. The authors identified 1714 records from Ovid EMBASE, Ovid Medline, Ovid Global Health, Web of Science, and Cochrane databases from January 2000 to August 2020. Studies that simply listed a few limited preselected comorbidities without any indication of clustering or grouping were excluded. After multiple rounds of screening (Fig. 3), 91 studies were fully read, leaving 12 eligible studies.

## Results

The final 12 studies included came from varying HIC settings except for 2 conducted in China^28^^,^^29^ and adopted a range of different methods that varied in analytical statistical depth (Table 1). From the sample, the earliest study was conducted in 2012,^30^ with most of the remaining studies produced after 2015.

**Table 1:** Characteristics of all included studies

Study Title	First Author, y	Study Design	Setting	n	Study Aim	Data Source	Measures/Outputs
Combination groupings							
Global health care use by patients with type 2 diabetes: does the type of comorbidity matter?	Calderón-Larrañaga et al,^40^ 2015	Longitudinal retrospective cohort study; negative binomial regression	Spain	65,716	To identify patterns of health care use among T2DM patients with multimorbidities	Primary care EHRs and the Hospital Minimum Basic Dataset (CMBD in Spanish)	IRR for health care use outcomes for each level of care: primary (no. of visits), specialist (no. of visits, no. of specialities visited), hospital (total and unplanned admissions, length of stay), emergency (no. of visits, no. of priority visits)
Comorbidity Burden and Health Services Use in Community-Living Older Adults with Diabetes Mellitus: A Retrospective Cohort Study	Gruneir et al,^39^ 2016	Retrospective cohort study	Canada	448,736	To examine comorbidity and its association with various health services among community-dwelling older adults with T2DM	Multiple linked population-based administrative databases (demographic, hospital, ambulatory, insurance, medication, home care)	Prevalence of number, type, and combinations (of 1, 2, 3) of comorbidities Frequencies of 1-y use of health services (physician visits, emergency department visits, inpatient hospital admissions, home care use, nursing home admissions)
Prevalence and coprevalence of comorbidities among patients with type 2 diabetes mellitus	Iglay et al,^37^ 2016	Retrospective cohort study	USA	1,389,016	To quantify the prevalence and coprevalence of comorbidities among T2DM patients	Quintiles electronic medical record	Prevalence and coprevalence of comorbidities
Multiple chronic conditions in type 2 diabetes mellitus: prevalence and consequences	Lin et al,^38^ 2015	Cross-sectional study; logistic regression	USA	161,174	To examine multiple chronic comorbidity (MCC) patterns among T2DM patients and identify comorbidity clusters associated with poor patient outcomes	Deidentified EHRs dataset from health care informatics company, Optum Humedica	Prevalence of MCC clusters among younger and older than age 65 y Predicted probabilities for diabetes outcomes (face-to-face visits, reaching glycated hemoglobin <8%) and health care outcomes (emergency visits, 30-d hospital readmission), for top 15 clusters
Prevalence and incidence density rates of chronic comorbidity in type 2 diabetes patients: an exploratory cohort study	Luijks,^30^ 2012	Longitudinal exploratory cohort study	Netherlands	714	To establish comorbidity rates in a primary-care population of patients with T2DM	Primary-care network: Continuous Morbidity Registration	Prevalence and incidence density rates of comorbidities and their clusters before, during, and after diabetes diagnosis (post-1, 5, 10 y)
Statistical clustering							
Multimorbidity in people with type 2 diabetes in the Basque Country (Spain): prevalence, comorbidity clusters, and comparison with other chronic patients	Alonso-Morán et al,^31^ 2015	Retrospective cohort study; agglomerative hierarchical clustering, logistic regression	Spain	1,473,937	To compare multimorbidity among patients with and without T2DM and identify disease clusters in T2DM patients	Primary care EHRs and the Hospital Minimum Basic Dataset (CMBD in Spanish)	Prevalence of no. of comorbidities and probabilities of having at least 1 comorbidity by age/sex/deprivation OR of having each of 51 chronic comorbidities with T2DM, and morbidity clusters of concurrently occurring conditions
Comorbidity in Adult Patients Hospitalized with Type 2 Diabetes in Northeast China: An Analysis of Hospital Discharge Data from 2002 to 2013	Chen et al,^28^ 2016	Longitudinal retrospective cohort study; hierarchical clustering	China	4,400,892	To evaluate comorbidity burden and patterns among hospitalized T2DM patients	EHR database	Prevalence of comorbidities by age/sex ACoR and RCoR of comorbidities co-occurring with T2DM. Clusters of comorbidities based on RCoRs
Latent class analysis suggests 4 classes of persons with type 2 diabetes mellitus based on complications and comorbidities in Tianjin, China: a cross-sectional analysis	Gao et al,^29^ 2017	Cross-sectional study; latent class analysis, multinomial logistic regression	China	5500	To classify persons with T2DM based on complications and comorbidities	Structured questionnaire of patients across 10 hospitals	Predicted probabilities of T2DM complications and comorbidities, to produce 4 classes OR of the association between class membership and various demographic factors, diabetes severity, and behavioral factors
Comorbidity network for chronic disease: a novel approach to understand type 2 diabetes progression	Khan et al,^34^ 2018	Health informatics study; graph theory and social network analysis	Australia	749,000	To understand the comorbidity pattern of T2DM and develop a research framework to model chronic disease progression in terms of comorbidity	Administrative private health care dataset with *ICD-10* diagnosis codes on hospital admission and discharge data	Modularity score and network node strength to produce comorbidity network of T2DM
The comorbidity burden of type 2 diabetes mellitus: patterns, clusters, and predictions from a large English primary care cohort	Nowakowska et al,^24^ 2019	Longitudinal retrospective cohort study; agglomerative hierarchical clustering	England	102,394	To quantify comorbidity patterns in people with T2DM, to estimate the prevalence of 6 chronic conditions in 2027 and to identify clusters of similar conditions	UK Clinical Practice Research Datalink health dataset linked with Index of Multiple Deprivation data	Crude and age-standardized prevalence of comorbidities, and their clusters at T2DM diagnosis and 2, 5, 9 y after diagnosis, by sex and deprivation
Differential Health Care Use, Diabetes-Related Complications, and Mortality Among Five Unique Classes of Patients with Type 2 Diabetes in Singapore: A Latent Class Analysis of 71,125 Patients	Seng et al,^33^ 2020 Solomon et al,^32^ 2017	Retrospective cohort study; latent class analysis, negative binomial, and Cox regressions	Singapore	71,125	To segment T2DM patients into distinct classes and evaluate their differential health care use, complications, and mortality patterns	Ministry of Health administrative database	5 classes of patients produced Frequencies of comorbidities by demographic factors. IRR of complications, health care use outcomes, and 4-y all-cause mortality among the classes
Identifying Subgroups of Type II Diabetes Patients using Cluster Analysis		Retrospective cohort study; hierarchical clustering using DIANA	USA	6250	To uncover correlations between demographic subgroups of T2DM patients and their comorbidities, among a predominantly African American population	Inpatient EHRs from Howard University	Comorbidity clusters of 2, 3, 4, 5 by distinct patient group characteristics based on sex and marital status

*Abbreviations:* ACoR, absolute co-occurrence risk; DIANA, divisive analysis; EHR, electronic health record; GP, general practice; *ICD*, *International Classification of Diseases*; ID, incidence density; IRR, incidence rate ratio; MCC, multiple chronic comorbidities; OR, odds ratio; RCoR, relative co-occurrence risk.

As the format of the study results was heterogenous, owing to the varied methods used, the authors have summarized studies by 2 broad categories: (a) those employing combination groupings of co-occurring conditions (ie, pairs and triads) to define multimorbidity (Table 2); and (b) those studies of more complex statistical clustering methods (Table 3). The key clustering approaches used in the reviewed studies included agglomerative^24^^,^^31^ and divisive^32^ hierarchical clustering,^28^ latent class analysis,^29^^,^^33^ and graph theory,^34^ all of which require substantially large health data sets. Moreover, novel machine learning approaches have been used to characterize and learn multimorbidity patterns,^35^^,^^36^ but these methods are yet to be applied to T2DM specifically. Although the study approaches and settings were varied, predictors and predominant groups of conditions across clusters were identified and are discussed in the following sections.

**Table 2:** Summary of results from studies adopting combination groupings

Study Title	First Author, y	Grouping Method	Grouping Pattern	Key Quantitative Outcomes
Global health care use by patients with type 2 diabetes: does the type of comorbidity matter?	Calderón-Larrañaga et al,^40^ 2015	EDC used^a^	4 mutually exclusive groups: 1. Individuals with no chronic comorbidities 2. Individuals with only concordant comorbidities 3. Individuals with at least 1 discordant physical comorbidity excluding those with mental comorbidities 4. Individuals with at least 1 mental comorbidity	The mean number of chronic comorbidities was higher in patients with mental comorbidity (5.5) compared with those with discordant physical comorbidity (4.1) or only concordant comorbidity (1.7) Within the groups of patients with discordant and mental comorbidity, 85.3% and 87.1% had at least 1 concordant comorbidity and 2.8% and 1.3% had >3 concordant comorbidities, respectively
Comorbidity Burden and Health Services Use in Community-Living Older Adults with Diabetes Mellitus: A Retrospective Cohort Study	Gruneir et al,^39^ 2016	EDC used^a^	Top 3 pairs of comorbid conditions: 1. Arthritis + hypertension (20.0%) 2. Other CVD conditions + hypertension (10.6%) 3. Disorders of lipid metabolism + hypertension (10.0%) Top 3 triads of comorbid conditions: 1. Arthritis + other CVD conditions + hypertension (9.9%), 2. Arthritis + disorders of lipid metabolism + hypertension (7.1%) 3. Arthritis + anxiety + hypertension (6.2%)	>90% of both men and women had at least 1 comorbid condition and more than 40% had 5+ conditions Hypertension was most common condition across all age groups, affecting 79.1% of the entire cohort. Arthritis affected 59.6% of the cohort, and other CVD conditions affected 59.3% 3 additional conditions affected more than one-third of the cohort: ischemic heart disease (37.6%), anxiety (36.9%), and disorders of lipid metabolism (33.7%)
Prevalence and co-prevalence of comorbidities among patients with type 2 diabetes mellitus	Iglay et al,^37^ 2016	Ranking of prevalence and coprevalence of comorbidities, assessed using *ICD-9-CM*	Top 5 pairs of comorbid conditions: 1. Hypertension and hyperlipidemia (67.5%) 2. Obesity and hypertension (66.0%) 3. Obesity and hyperlipidemia (62.5%) 4. Hypertension and CKD (22.4%) 5. Hyperlipidemia and CKD (21.1%)	In those <65 y of age, only 12.4% of patients had CKD, but this increased to 27.7% in those 65–74 y and 43.2% in those 75+ y Due to the higher prevalence of obesity in those aged <65, obesity and hypertension (66.1%) were the most common comorbidity pair For patients aged 65+, the highest coprevalence was for the combination of hypertension and hyperlipidemia (65–74 y: 76.2%; 75+ y: 75.2%)
Multiple chronic conditions in type 2 diabetes mellitus: prevalence and consequences	Lin et al,^38^ 2015	Prevalence-driven groupings: prevalence rate of each comorbid condition examined, and *t* tests used to compare prevalence between patients aged <65 and ≥65 y. For analysis of MCCs, the most prevalent comorbidities that affected ≥5% of the sample were prioritized	Ranked combinations: 1. Obesity-hyperlipidemia-hypertension (19%) 2. Hyperlipidemia-hypertension (17%) 3. Obesity-hyperlipidemia (4%) 4. Obes-hyperlipidemia-hypertension-CAD (2.5%) 5. Hyperlipidemia-hypertension-CAD (2.5%) 6. Obesity-hyperlipidemia-hypertension-COPD/asthma (1.5%) 7. Obesity-hyperlipidemia-hypertension-Arthritis (1.5%)	Overall, 51% had some combination of hypertension, hyperlipidemia and obesity Clusters including obesity were far more common among younger adults (aged <65 y); the leading MCC cluster was hypertension-hyperlipidemia-obesity (23%), whereas among older adults (aged ≥65 y), the most common cluster was hypertension and hyperlipidemia only (20%) 14% of younger patients had no diagnosed comorbidities, compared with 11% of older patients. Older patients exhibited greater cluster heterogeneity; the top 10 clusters accounted for a smaller proportion of older adults (66%) than younger adults (78%)
Prevalence and incidence density rates of chronic comorbidity in type 2 diabetes patients: an exploratory cohort study	Luijks et al,^30^ 2012	Comorbid diseases were classified into clusters, according to the diagnostic chapters of *ICPC-1*	Prevalent disease groups at T2DM diagnosis: Cardiovascular (64.0%) Musculoskeletal (31.1%) Mental (24.1%) Urogenital (15.4%) Respiratory (14.1%) Skin (9.9%)	15.4% of patients did not have a chronic comorbidity at diabetes diagnosis. 27.2% had 3+ discordant comorbidities Ear and eye diseases (particularly cataract) had a high incident density rate after diabetes diagnosis compared with before diagnosis 46.9 per 1000 patient-years 26.4% of those without any chronic condition at time of diabetes diagnosis developed at least 1 comorbidity in the first subsequent year

*Abbreviations:* CAD, coronary artery disease; COPD, chronic obstructive pulmonary disease; CVD, cardiovascular disease; EDC, expanded diagnostic clusters; *ICD-9-CM*, *International Classification of Diseases, Ninth Revision, Clinical Modification*; *ICPC-1*, *International Classification of Primary Care, First Version*; MCC, multiple chronic comorbidities.

a The EDC is internationally validated and groups *ICPC* and *ICD* codes into 260 clusters based on clinical, diagnostic, and therapeutic similarities.

**Table 3:** Summary of results from studies adopting statistical clustering

Study Title	First Author, y	Clustering Method	Clustering Pattern	Key Quantitative Outcomes
Multimorbidity in people with type 2 diabetes in the Basque Country(Spain): prevalence, comorbidity clusters and comparison with other chronic patients	Alonso-Morán et al,^31^ 2015	Agglomerative hierarchical clustering (Ward's minimum-variance method)	Cluster A: concordant diseases directly T2DM-related (hypertension, IHD, AF, other chronic heart diseases, CKD, heart failure) Cluster B: mental illnesses (anxiety and depression) Cluster C: digestive, bone and joint diseases (dyspepsia, degenerative joint disease, low back pain, osteoporosis, diverticular intestine disease, rheumatoid arthritis, autoimmune and connective tissue diseases) Cluster D: vision diseases (glaucoma, blindness, low vision) Cluster E: respiratory diseases (asthma, emphysema, chronic bronchitis, COPD)	OR of the comorbidity co-occurrence with T2DM: PVD (2.24), heart failure (2.0), hypertension (1.97), CKD (1.83), transplant status (1.75), chronic liver disease (1.65) Prevalence of having: 1+ comorbidity (89.7% overall, [87.6% of men, 92% of women]); 3+ comorbidities (46.5%); 10+ comorbidities (1.7% of men, 1.9% of women)
Comorbidity in Adult Patients Hospitalized with Type 2 Diabetes in Northeast China: An Analysis of Hospital Discharge Data from 2002 to 2013	Chen et al,^28^ 2016	Hierarchical clustering (Ward’s minimum-variance method with Euclidean distance measure) ACoR^a^ and RCoR^b^ calculated for each condition. Major T2DM-related comorbidities were defined as having both ACoR >1% and RCoR >1 27 major comorbidities clustered into 3 categories with high, medium, and low RCoRs with T2DM	a. High RCoR group: • Essential hypertension: peripheral & visceral atherosclerosis • Disorders of lipid metabolism: chronic renal failure • Occlusion or stenosis of precerebral artery: urinary tract infections • Other endocrine disorders: coronary atherosclerosis • Other cerebrovascular diseases: acute myocardial infarction • Other nutritional, endocrine, & metabolic disorders b. Medium RCoR group • Acute cerebrovascular disease: skin & subcutaneous tissue infection • Other liver diseases: fluid & electrolyte disorders • Conduction disorders: other upper respiratory infections • Other lower respiratory disease: pneumonia • Congestive heart failure: transient cerebral ischemia • Acute bronchitis c. Low RCoR group: • Cardiac dysrhythmias: hyperplasia of prostate • Noninfectious gastroenteritis: other eye disorders • Thyroid disorders: biliary tract disease • Other nervous system disorders: cataract	Overall, essential hypertension was the most common comorbidity with ACoR of 58.4%, whereas peripheral and visceral atherosclerosis had the strongest association with T2DM with RCoR of 4.206 Dyslipidemia was the second most severe comorbidity with RCoR of 3.447 (median RCoR: men [2.779], women [2.099]). Women had higher co-occurrence of chronic renal failure (RCoR 2.461) than men (RCoR 2.155) Older patients (aged 60–69) had the highest prevalence of comorbidities with hypertension (32.7%), CHD (28.5%), acute CVD (25.9%) being top conditions
Latent class analysis suggests 4 classes of persons with type 2 diabetes mellitus based on complications and comorbidities in Tianjin, China: a cross-sectional analysis	Gao et al,^29^ 2017	LCA, multinomial logistic regression	Class 1: complications & comorbidities group (6.1%); high conditional probability of suffering from all complications and comorbidities. Highest age, family history, central obesity, and suburban residence Class 2: high risk of complications group (25.7%); diabetic peripheral neuropathy, retinopathy, and lower-limb vascular disease Class 3: high risk of comorbidities and CVD group (14.3%): hypertension, dyslipidemia and metabolic syndrome, and CVD Class 4: diabetes without complications & comorbidities group (53.9%): low conditional probabilities for all complications. Youngest of the groups, lowest family history, female proportion, and central obesity	Overweight (46.9%) and obesity (21.3%) were prevalent among T2DM patients. Patients with higher BMIs had higher odds of being in class 1 (OR 1.43) and class 3 (OR 1.45) Women aged <48.5 y had lower adjusted odds of being in class 2 (OR 0.59) and class 3 (OR 0.46), compared with men. However, women aged 48.5+ y had higher adjusted odds of being in class 3 (OR 1.40)
Comorbidity network for chronic disease: a novel approach to understand type 2 diabetes progression	Khan et al,^34^ 2018	Network theory, social network analysis Disease networks created with directional edges based on admission sequence, with varying node strengths.^c^ An overall comorbidity network created based on the trajectory of the population cohort over time, and the unique characteristics of progression toward T2DM vs non-T2DM patients. Generated by aggregating individual disease networks	Cluster 1: CVD-related diseases (hypertension, cardiac arrhythmias, presence of bypass grafts, COPD) along with cancer and anemia-related conditions Cluster 2: liver disease, long-term insulin use, behavioral disorders (depression, psychoses, drug abuse) Cluster 3: other heart-related comorbidities (congestive heart failure, coronary angioplasty implants) and pulmonary circulation disorders	The node strengths of top comorbidities and conditions in descending order were as follows: cardiac arrhythmias (1), long-term use of insulin (1), liver disease (0.35), cataract (0.25), valvular disease (0.15), uncomplicated hypertension (0.125), presence of aortocoronary bypass graft (0.125), presence of coronary angioplasty implant and graft (0.12), congestive heart failure (0.11), pulmonary circulation disorders (0.1)
The comorbidity burden of type 2 diabetes mellitus: patterns, clusters, and predictions from a large English primary care cohort	Nowakowska et al,^24^ 2019	Agglomerative hierarchical clustering, prevalence at 0, 2, 5, 9 y after diagnosis of diabetes	At diagnosis: Cluster 1: PVD, CHD, stroke, atrial fibrillation, heart failure Cluster 2: cancer, hypertension, CKD Cluster 3: depression, SMI Cluster 4: COPD, asthma Cluster 5: hypothyroidism, rheumatoid arthritis, osteoporosis 2-yafter diagnosis: Cluster 1: hypertension, cancer, rheumatoid arthritis, osteoporosis, hypothyroidism Cluster 2: CKD, atrial fibrillation, heart failure, PVD, CHD, stroke, dementia Cluster 3: COPD, asthma, depression, SMI 5-y after diagnosis: Cluster 1: hypothyroidism, osteoporosis, rheumatoid arthritis, dementia, stroke Cluster 2: CKD, hypertension, cancer Cluster 3: CHD, PVD, heart failure, atrial fibrillation Cluster 4: SMI, depression, asthma, COPD 9-y after diagnosis: Cluster 1: CHD, atrial fibrillation, heart failure, PVD, COPD Cluster 2: SMI, depression, asthma Cluster 3: hypothyroidism, rheumatoid arthritis, osteoporosis Cluster 4: hypertension, CKD, cancer, stroke, dementia	Hypertension and CKD had the highest age-standardized coprevalence rate among all T2DM patients: 12.1% at the time of T2DM diagnosis, and 15.4%, 17.8%, and 21.5% after 2, 5, and 9 y after diagnosis. Overall, the second most coprevalent combination at diagnosis is hypertension and CHD at 9.3% Depression prevalence increased over the study period for all T2DM patients. Age-standardized prevalence for women was higher than men at both time points in 2007 (women: 15.9%, men: 7%) and in 2016 (women: 21.5%, men: 10.4%)
Differential Health Care Use, Diabetes-Related Complications, and Mortality Among Five Unique Classes of Patients With Type 2 Diabetes in Singapore: A Latent Class Analysis of 71,125 Patients	Seng et al,^33^ 2020	LCA used to derive groups of homogenous individuals by age (ie, less than [younger] and >65 [older]), ethnicity, duration of diabetes, and comorbidities. Cox regression models used to ascertain the relationship between class membership and risk of complications	Class 1: younger patients with short T2DM duration and “relatively healthy” (15.7%) Class 2: younger patients with short to moderate T2DM duration and moderate disease burden without end-organ complications (34.5%) Class 3: younger women with short to moderate T2DM duration and high psychiatric and neurologic disease burden (1.58%) Class 4: older patients with moderate T2DM duration and moderate disease burden (36.9%) Class 5: older patients with moderate to long T2DM duration, with depression, dementia, and high disease burden with end-organ complications (11.3%) *T2DM duration: short (<5 y), moderate (5–10 y), long (>10 y)*	Prevalence of key conditions are expressed as bands, mapped by class >100% prevalence: Class 3: anxiety, general anxiety disorder, major depression, schizophrenia, bipolar disorder, hemorrhagic stroke, dementia Class 5: major depression, CHD, previous MI, coronary artery bypass graft, PCI, heart failure, ESRD, kidney transplant, stroke, dementia, PVD, LEA >50%–100% prevalence: Class 4: dementia 25% ≤ 50% prevalence: Class 1: kidney transplant Class 3: stroke, ischemic stroke
Identifying Subgroups of Type II Diabetes Patients using Cluster Analysis	Solomon et al,^32^ 2017	Hierarchical clustering using DIANA to produce clusters of 2, 3, 4, and 5, by sample subgroups	For clusters of 5 (ie, considers all the patient segments in the sample): Cluster 1: (single, male): hypertension, hyperlipidemia, tobacco use disorder, onychomycosis, cholesterolemia, dehydration Cluster 2: (nonsingle, male): hyperlipidemia, hypertension, onychomycosis, cholesterolemia, malignant neoplasm of prostate, benign neoplasm of colon Cluster 3: (single, female): hypertension, hyperlipidemia, cholesterolemia, onychomycosis, tobacco use disorder, benign neoplasm of colon Cluster 4: (nonsingle, female): hyperlipidemia, hypertension, cholesterolemia, benign neoplasm of colon, onychomycosis, acquired hypothyroidism Cluster 5: (married, both sexes): hypertension, hyperlipidemia, benign neoplasm of colon, cholesterolemia, ergosterol deficiency, onychomycosis	The top conditions across all the clusters and subgroups for both sexes were hypertension, hyperlipidemia, and cholesterolemia, with similar prevalence Hypertension: prevalence in single men (9.6%), nonsingle men (10.4%), single women (9%), nonsingle women (9.6%), married both (11.6%) Hyperlipidemia: prevalence in single men (8.7%), nonsingle men (11.2%), single women (8.6%), nonsingle women (9.8%), married both (8.96%) Cholesterolemia: prevalence in single men (3.7%), nonsingle men (4.3%), single women (4.6%), nonsingle women (5.3%), married both (4.5%)

*Abbreviations:* AF, atrial fibrillation; BMI, body mass index; ESRD, end-stage renal disease; IHD, ischemic heart disease; LCA, latent class analysis; LEA, lower-extremity amputation; MI, myocardial infarction; PCI, percutaneous coronary intervention; SMI, severe mental illness.

a ACoR (%) was calculated as a proportion of the occurrence of a comorbidity over the total occurrence of both the comorbidity and T2DM in the population.

b RCoR was calculated as the ratio of the occurrence of a comorbidity in those with T2DM over occurrence in those without T2DM, in the population.

c Node strength shows relative attribution of the comorbidity/condition for the most hospital admissions for diabetic patients in the final comorbidity network constructed.

### Who Is at Risk of Type 2 Diabetes Mellitus–Related Multimorbidity?

Age and deprivation are the leading drivers of multimorbidity, as the prevalence and number of comorbidities increase with older age in patients with T2DM.^28^^,^^33^^,^^37^ For example, the proportion of those with “complex” multimorbidity, defined as 4+ comorbidities, increases from 25% among persons less than 65 years to 42% among those aged 65 to 74 years, and 49% among those 75+ years of age.^37^ Patients older than 65 years and living with T2DM for 10+ years had a higher multimorbidity burden, characterized by end-organ complications, such as myocardial infarction and end-stage renal disease.^33^ Interestingly, clusters containing obesity were much more common in younger adults compared with those older than 65 years.^38^ Increasing deprivation level was strongly and consistently associated with an increasing proportion of patients with T2DM and a greater number of concurrent comorbidities^31^; the age-standardized prevalence of 1 or more comorbid conditions was 33.3% for the least-deprived areas and 32.7% for the most-deprived areas.^24^ For 4 or more chronic conditions, the age-standardized prevalence was 2.9% in the most affluent areas and 4.4% in the most deprived areas.^24^

Women have a proportionally greater burden of multimorbidity than their male counterparts in some studies,^24^^,^^29^^,^^31^^,^^33^ although the magnitude of this association is modest and inconsistent. A greater proportion of women presented with 4+ co-occurring comorbidities than men (18% vs 15.9%),^31^ with postmenopausal women experiencing the greatest risk of developing further comorbidities.^29^ However, a US study found that although more women had 1 or 2 comorbidities, more men had 3 or 4 comorbidities.^37^ In addition, there was little effect of sex on comorbidity number among patients aged 65+.^39^

Studies of T2DM-related multimorbidity clustering have not yet examined the relationship with common physiologic risk factors commonly associated with T2DM complications, such as elevated HbA1c, blood pressure, and levels of obesity.

### How Are Type 2 Diabetes Mellitus–Related Multimorbidity Clusters Characterized?

Most reviewed studies were cross-sectional in nature, characterizing multimorbidity among patients at a specific point in time, with some studying patients with varying duration of T2DM. Across studies, 3 types of condition clusters appeared consistently, namely, (a) cardiometabolic precursor conditions, (b) vascular conditions, and (c) mental health conditions.

Across 6 diverse study settings, cardiometabolic precursor conditions, such as disorders of lipid metabolism, obesity, and hypertension, were the most common conditions at diagnosis.^24^^,^^32^^,^^37^, ^38^, ^39^ Hypertension was consistently the most common condition in multimorbid combinations and clusters.^24^^,^^31^^,^^32^^,^^37^, ^38^, ^39^ In individuals with 2 comorbid conditions in addition to T2DM, hypertension was commonly found alongside hyperlipidemia (67.5%),^37^ arthritis (20%), and anxiety (6.8%).^37^^,^^39^ Among individuals with 3 conditions in addition to T2DM, 51% had some combination of hypertension, hyperlipidemia, and obesity.^38^

The authors identified similar clusters of conditions that predominantly contained concordant cardiovascular and microvascular conditions usually associated with later-stage disease, such as coronary heart disease (CHD), stroke, atrial fibrillation, peripheral vascular disease (PVD), and chronic kidney disease (CKD).^24^^,^^28^^,^^29^^,^^31^ Cardiovascular conditions were the most prevalent comorbidity type at diagnosis (64%)^30^ and constituted the second most prevalent triad alongside hypertension.^39^ These results were consistent across different clustering methodologies, with a study applying novel graph theory techniques to hospital admissions finding a distinct cluster of cardiovascular-related conditions, including cardiac arrythmias and CHD.^34^ Three conditions, namely hypertension, CHD, and acute cerebrovascular disease, formed a comprehensive multimorbid web with T2DM.^28^ These conditions were the most common comorbidities of T2DM, and conversely, the other way around whereby T2DM was also the most common comorbidity of each of these conditions.^28^^,^^30^ This web demonstrates a bidirectional, mutually reinforcing association between these conditions and T2DM.

Several studies exhibited distinct clusters containing mainly mental health conditions.^24^^,^^31^^,^^33^^,^^34^ In an English cohort, a cluster composed of depression, severe mental illness, chronic obstructive pulmonary disease, and asthma was observed regardless of duration lived with T2DM,^24^ and with more prominent representation among women.^24^^,^^33^ Specifically, Seng and colleagues^33^ found that younger women formed a distinct cluster with high psychiatric disease burden, including anxiety, major depression, schizophrenia, and bipolar disorder, whereas an Australian study found a similar cluster predominantly containing conditions such as depression, psychoses, and substance abuse, along with liver disease.^34^

### How Does Multimorbidity Vary According to Duration of Type 2 Diabetes Mellitus?

**Fig. 4:**
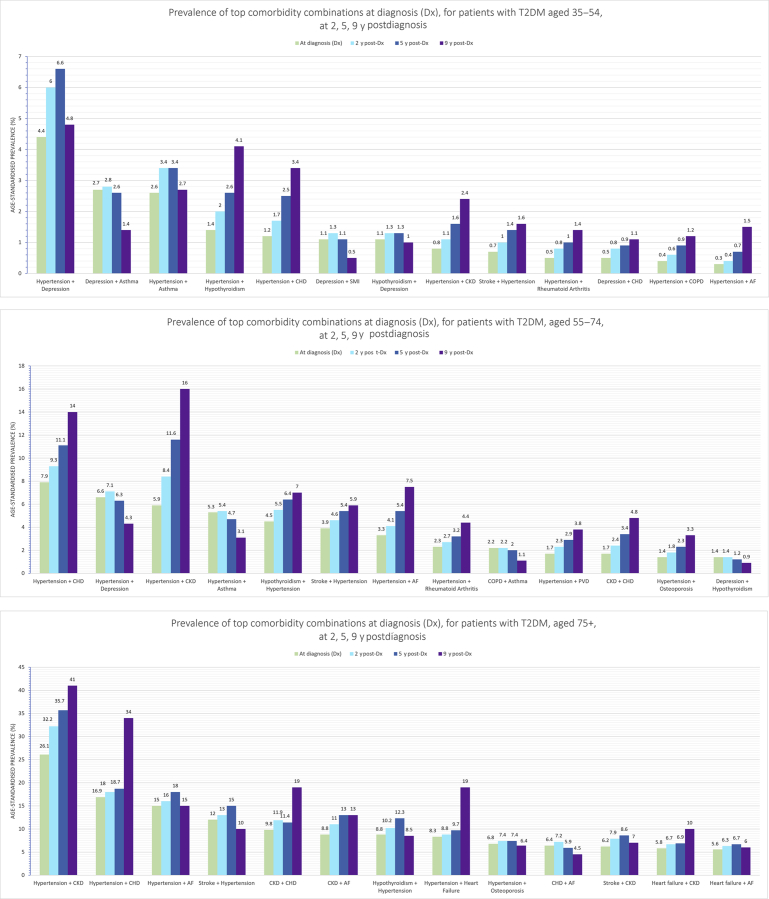
Prevalence of top comorbidity combinations at diagnosis for patients with T2DM. Prevalence of top comorbidity combinations at diagnosis (Dx), for patients with T2DM at 2, 5, 9 years after diagnosis for 3 age bands. AF, atrial fibrillation; COPD, chronic obstructive pulmonary disease; SMI, severe mental illness.

There was a clear lack of longitudinal cohort-based multimorbidity studies that tracked specific patients over time. Rather, the few studies that considered some element of time stratified patients by the duration lived of T2DM at the time of study. A cluster of older patients with moderate to long T2DM duration were found to have high disease burden with end-organ complications, for example, myocardial infarction, end-stage renal disease, stroke, and amputation.^33^ This finding was consistent with findings by Gao and colleagues,^29^ as longer T2DM duration and older age were associated with a higher prevalence of all comorbidities. Cluster analysis at 2, 5, and 9 years post-T2DM diagnosis revealed a diversification of conditions as T2DM progresses.^24^ This finding was demonstrated by an increase in the number and heterogeneity of clusters formed, departing from the previously distinct body systems found in clusters of earlier duration, for example, closer clustering of depression and asthma at 9 years after diagnosis compared with at diagnosis.^24^ Hypertension consistently appeared in the top combination duos of conditions at both 2 and 5 years after diagnosis of T2DM alongside CKD (15.4%, 17.8%), CHD (10.3%, 11.3%), atrial fibrillation (7.5%, 8.7%), stroke (6.9%, 8.0%), and across age stratifications (Fig. 4).^24^ Interestingly, out of all the chronic comorbidities, hypertension had the highest incidence density in the year before T2DM diagnosis (75.2 new cases per 1000 patient-years), but decreased significantly at 5 years after diagnosis (42.6 new cases per 1000 patient-years).^30^

(*Adapted from* Nowakowska M, Zghebi SS, Ashcroft DM, et al. The comorbidity burden of type 2 diabetes mellitus: patterns, clusters and predictions from a large English primary care cohort. BMC Med 2019;17(1):145; with permission.)

### What Is the Impact on Health Care Utilization?

Patients with diabetes have high rates of hospitalization, more complex hospital trajectories, with more transitions between other conditions, and double the number of “admission sequences” compared with patients without diabetes.^34^ Moreover, rates of health care utilization are disproportionately higher in T2DM patients with mental health comorbidities than those without.^33^^,^^40^ Seng and colleagues^33^ found that the use of tertiary health care was highest among (a) “younger females with short to moderate T2DM duration and high psychiatric disease burden” and (b) “older patients with moderate to long T2DM duration and high disease burden with end-organ complications.”^33^ Compared with healthy patients, the risk of emergency department visits was 3.31 times higher for group (a) and 2.47 times higher for group (b).

Having a mental comorbidity was significantly and independently associated with a 1.25 times increased risk of being admitted to hospital, 1.30 times as likely to use emergency and specialist care, and 1.17 times as likely to visit the general practitioner compared with only having physical concordant comorbidities.^40^ This finding highlights the amplified effect of mental comorbidities on health care utilization for multimorbid patients with T2DM.

Overall, the reviewed studies demonstrated that:

• The multimorbidity clusters were dominated by conditions directly related to T2DM pathogenesis, for example, hypertension, disorders of lipid metabolism, and obesity, often considered risk factors for other comorbidities.^24^^,^^28^^,^^31^^,^^32^^,^^37^^,^^38^
• Across several studies, concordant vascular-related conditions frequently appeared in clusters^24^^,^^28^^,^^29^^,^^31^ and combination groupings,^30^^,^^37^^,^^38^ such as CHD, PVD, stroke, and CKD.
• Four studies demonstrated distinct clusters of mental health comorbidities, such as depression and anxiety,^31^^,^^34^ with particularly elevated prevalence among younger women.^24^^,^^33^
• As the duration of T2DM increases, clusters demonstrate increased heterogeneity departing from clusters previously belonging to distinct body systems, which indicates diversification of multimorbidity at progressive intervals.^24^
• Health care utilization rates are high among multimorbid patients with T2DM,^34^ especially among those with mental health comorbidities.^33^^,^^40^

## Discussion

In this first-ever review of the findings of epidemiologic studies focusing on T2DM-related multimorbidity clustering, the authors found common comorbidities of T2DM to be hypertension, cardiovascular-related diseases, microvascular conditions, depression, and lipid disorders. Their findings indicate that generally T2DM individuals who are older, female, and more deprived are at a higher risk of having T2DM-related multimorbidity.

Concordant comorbidities, such as hypertension and cardiovascular and microvascular conditions, dominated clusters across the studies, agreeing with global trends in T2DM complications^27^ and novel nonclustering studies.^41^ Hypertension was the most central condition, indicating it was an important mediator of connections between other conditions, seen across various subgroups.^41^ This finding is consistent with the long-standing literature that hypertension is the key driver in the development of cardiovascular and microvascular conditions. Given the highly prevalent co-occurrence of hypertension and other precursor conditions, such as obesity and lipid disorders, and their established roles as drivers of vascular morbidity and mortality, it is important to focus on these as key risk factors in the control and management of T2DM and related multimorbidity. In the case of PVD, prolonged time spent with hypertension can worsen downstream microvascular conditions, such as retinopathy and foot ulcers, which are often irreversible and should be the focus of secondary prevention programs.^28^

Although concordant comorbidities, such as hypertension and hyperlipidemia, are central to T2DM-related multimorbidity, discordant comorbidities, such as depression and osteoarthritis, can have a direct adverse effect on self-care behaviors.^26^ Specifically, mental health disorders may have effects on T2DM self-management, quality of life, and subsequent control of overlapping risk factors, such as diet and physical activity.^42^ As these findings indicate, depression is frequently associated with T2DM-related multimorbidity consistent with epidemiologic research that demonstrates the increasing prevalence of comorbid depression in T2DM populations globally.^27^^,^^43^^,^^44^ The association of T2DM with depression is likely to be bidirectional and involving common hormonal and inflammatory pathways.^45^ The concurrence of both physical conditions and mental health disorders has been associated with significantly worse health outcomes, compared with those without comorbid mental illness^42^; this indicates a need to adopt a more holistic approach to managing T2DM-related multimorbidity, such that deterioration in mental health can be detected early to prevent further morbidity burden.

### Challenges Within the Field of Type 2 Diabetes Mellitus–Related Multimorbidity

The identification of multimorbidity clusters serves several purposes. First, it allows for the descriptive characterization of multimorbidity in populations in a way that takes into account a comprehensive spectrum of conditions; second, it identifies subsets of the population that may be driving demands on health systems; and third, it provides clues to the diverse etiologic pathways of T2DM-related morbidity. However, this review exposes several challenges and gaps in this rapidly evolving literature on T2DM-related multimorbidity.

First, the current literature does not provide clear distinction of the temporality or sequence of phenotypes of multimorbidity and clustering. Specific temporal trends in T2DM-related multimorbidity are yet to be studied in more population-based settings,^28^ as reviewed studies were largely cross-sectional. In addition, concordant conditions are likely to have an inflated coprevalence with T2DM because of care-related factors; for example, clinicians will be more attentive for T2DM in a patient that has myocardial infarction.^30^ Second, it is difficult to infer the magnitude of association and risk of different forms of multimorbidity. Traditional definitions of multimorbidity rely on counts of conditions^18^ and do not normally consider important factors, such as the burden of the disease, severity, and the interaction between conditions, as this is reflected in most of the reviewed studies that predominantly report on counts or prevalence. Third, the heterogeneity of methodologic approaches, data inputs, and condition classifications makes comparisons across studies difficult. Cluster analysis itself is an exploratory classification method, and different algorithms can yield different results.^31^ These limitations have made for difficult interpretation, and translation of findings into meaningful clinical applications.

### Future Direction: Longitudinal Clustering

To make this literature more useful and interpretable for clinical and public health practice, adoption of specific reporting guidelines across cluster analyses^46^; the application of such techniques to a variety of low-income settings; and the advancement of clustering research toward more longitudinal progression-focused exploratory approaches utilizing the advances of machine learning should be considered.^35^ A shift from a prevalence-based to an incidence-based epidemiologic approach guided by etiologic medical literature will reveal the trajectories of multimorbidity rather than patterns conflated by risk factors accrued earlier in the patient journey. Such approaches are crucial to reveal temporal associations, to estimate population preventable fractions, and to identify leverage points whereby an intervention can have a greater magnitude of effect in multimorbid patients compared to a more downstream intervention.

### Clinical Applications: Stratification and Holistic Care

As further work identifies risk patterns for multimorbidity among subpopulation groups, T2DM populations could be stratified according to the risk of developing multimorbidity, dominant clusters of conditions in order to implement specific preventative measures. For instance, the authors’ findings show it is important to prioritize dementia screening for patients with longer T2DM duration, and depression, specifically for younger women.^24^^,^^33^ Alongside mental health conditions, identifying and managing other prominent comorbidities have the potential to reduce polypharmacy burden through control of common risk factors and to optimize treatment pathways via reductions in fragmented care.^10^^,^^40^ Targeting central conditions (eg, hypertension and obesity), themselves risk factors for a host of other conditions, can realign care provisions beyond a traditional single disease focus.

## Summary

T2DM-related multimorbidity is complex and exhibits variation in different study populations. Common comorbidities in patients with T2DM are hypertension, lipid disorders, cardiovascular-related conditions (eg, CHD), microvascular conditions, and depression. Patients with T2DM that are older, female, and more deprived are generally at higher risk of developing multimorbidity. Clinicians should be increasingly aware of the more heterogeneous needs of individuals living with T2DM, particularly surrounding mental health. Future research is needed to explore more granular patient trajectories, their associated risk factors, and population-level temporal trends to further both clinical delivery and public health planning purposes.

## Clinics care points

• *Patient stratification is key to tackling multimorbidity at the clinical level:* a preventative approach to detecting and monitoring specific comorbidities with high likelihood in particular demographic segments, for example, development of risk scores to guide timely intervention for delaying end-stage organ complications like renal failure or myocardial infarction in middle-aged patients with chronic kidney disease or cardiovascular disease, respectively.
• *Research to assess suitability of a depression and/or dementia screening program for patients with type 2 diabetes mellitus:* increased provision of screening for depression among younger adults, particularly women, and dementia for patients with longer type 2 diabetes mellitus duration is perhaps a crucial component in curbing the severity of type 2 diabetes mellitus–related multimorbidity.
• *Holistic and dynamic clinical treatment and management:* important to focus management of concordant type 2 diabetes mellitus comorbidities through common risk factors (eg, hypertension and hyperlipidemia) and account for patients’ behavioral risk factors (eg, exercise, diet, smoking) and living contexts (eg, the effect of deprivation) for more holistic care.

## Disclosure

J. Pearson-Stuttard is vice-chairman of the Royal Society for Public Health and reports personal fees from Novo Nordisk A/S and Lane Clark & Peacock LLP outside of the submitted work. All other authors have nothing to disclose. This work has been funded by the 10.13039/501100000265Medical Research Council (MRC) UK, grant number MR/V005057/1.
